# Ago1 Interacts with RNA Polymerase II and Binds to the Promoters of Actively Transcribed Genes in Human Cancer Cells

**DOI:** 10.1371/journal.pgen.1003821

**Published:** 2013-09-26

**Authors:** Vera Huang, Jiashun Zheng, Zhongxia Qi, Ji Wang, Robert F. Place, Jingwei Yu, Hao Li, Long-Cheng Li

**Affiliations:** 1 Department of Urology and Helen Diller Family Comprehensive Cancer Center, University of California San Francisco, San Francisco, California, United States of America; 2 Department of Biochemistry and Biophysics, University of California San Francisco, San Francisco, California, United States of America; 3 Department of Laboratory Medicine, University of California San Francisco, San Francisco, California, United States of America; Cincinnati Children's Hospital Medical Center, United States of America

## Abstract

Argonaute proteins are often credited for their cytoplasmic activities in which they function as central mediators of the RNAi platform and microRNA (miRNA)-mediated processes. They also facilitate heterochromatin formation and establishment of repressive epigenetic marks in the nucleus of fission yeast and plants. However, the nuclear functions of Ago proteins in mammalian cells remain elusive. In the present study, we combine ChIP-seq (chromatin immunoprecipitation coupled with massively parallel sequencing) with biochemical assays to show that nuclear Ago1 directly interacts with RNA Polymerase II and is widely associated with chromosomal loci throughout the genome with preferential enrichment in promoters of transcriptionally active genes. Additional analyses show that nuclear Ago1 regulates the expression of Ago1-bound genes that are implicated in oncogenic pathways including cell cycle progression, growth, and survival. Our findings reveal the first landscape of human Ago1-chromosomal interactions, which may play a role in the oncogenic transcriptional program of cancer cells.

## Author Summary

Argonaute (Ago) proteins are an evolutionarily conserved family of proteins indispensable for a gene regulation mechanism known as RNA interference (RNAi) which is mediated by small RNA including microRNA (miRNA) and small interfering RNA (siRNA) and occurs mainly in the cytoplasm. In mammalian cells, however, the function of Agos in the nucleus is largely unknown despite a few examples in which Agos are shown to be involved in regulating gene transcription and alternative splicing. In this study, by taking a genome-wide approach, we found that human Ago1, but not Ago2, is pervasively associated with gene regulatory sequences known as promoter and interacts with the core component of the gene transcription machinery to exert positive impact on gene expression in cancer cells. Strikingly, the genes bound and regulated by Ago1 are mostly genes that stimulate cell growth and survival, and are known to be involved in the development of cancer. The findings from our study unveil an unexpected role of nuclear Ago1 in regulating gene expression which may be important both in normal cellular processes and in disease such as cancer.

## Introduction

Argonautes (Ago) comprise a family of evolutionarily conserved proteins that are central to the RNA interference (RNAi) platform and miRNA function [1], [2]. Ago proteins are often recognized by their cytoplasmic function in which they regulate gene transcripts via post-transcriptional gene silencing (PTGS) mechanisms. However, nuclear functions have also been well-characterized in fission yeast and plants in which they assist in mechanisms of transcriptional gene silencing (TGS). In fission yeast, Ago partners with antisense transcripts to form the RITS (RNA-induced transcriptional silencing) complex at centromeric regions to induce heterochromatin formation [3]. Similarly, plant Argonautes interact with ribonucleoprotein complexes to induce histone and DNA methylation [4].

In mammals, the nuclear role of Ago proteins (Ago1–4) has remained largely unexplored. There have been scattered examples implicating mammalian Ago members in several nuclear processes including TGS [5]–[8], gene activation [9]–[11], and alternative splicing [12]. In the present study, we investigate the nuclear functions of Ago1 and Ago2 – the major facilitators of miRNA activity [13], [14] – from a global prospective using human cancer cells as a model system. Initial biochemical experiments indicate that nuclear Ago1 selectively interacts with RNA polymerase II (RNAP II). Chromatin immunoprecipitation coupled with massively parallel sequencing (ChIP-seq) reveals nuclear Ago1, but not Ago2, is pervasively associated with promoters of actively transcribed genes involved in growth, survival, and cell cycle progression. Ago1 knockdown experiments further indicate a positive correlation between Ago1 binding and gene expression. Additional evidence suggests that Ago1-chromosomal interactions may be dependent on miRNA. Our data represents the first landscape of Ago1-chromosomal interactions in human cells and reveals a novel function for Ago1 in modulating gene transcription within the nucleus.

## Results

### Nuclear localization and distribution of Ago1 and Ago2

**Figure 1:**
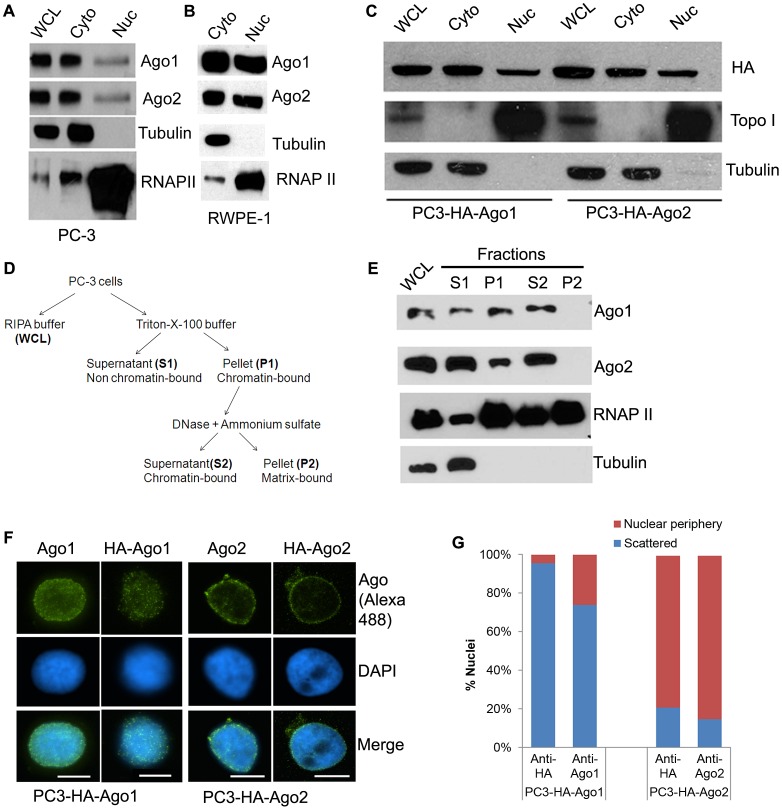
Differential nuclear localization of Ago1 and Ago2. (**A and B**) Protein levels of Ago1, Ago2, RNA Polymerase II (RNAP II), and tubulin were detected in whole cell lysate (WCL), nuclear (Nuc), or cytoplasmic extracts (Cyto) from PC-3 cells (A) and in Nuc or Cyto fractions from RWPE-1 cells (B) using protein-specific antibodies. RNAP II and tubulin served as nuclear and cytoplasmic markers, respectively. (**C**) Localization of HA-tagged Ago1 (HA-Ago1) and Ago2 (HA-Ago2) were determined in their respective stable cell lines by immunoblot analysis using an antibody specific to the HA epitope. (**D**) Depicted is the cell fractionation protocol for isolating chromatin-bound protein. (**E**) All fractions including WCL from PC-3 cells were evaluated by immunoblot analysis. RNAP II and tubulin served as markers for chromatin-bound and unbound protein, respectively. Factors bound to chromatin are found in fractions P1 and S2. (**F**) Purified nuclei from stable cell lines expressing HA-Ago1 (PC3-HA-Ago1) or HA-Ago2 (PC3-HA-Ago2) were analyzed by IF. Antibodies specific to Ago1 or Ago2 detected both endogenous and exogenous forms. An antibody specific to the HA epitope visualized only HA-Ago1 or HA-Ago2. DAPI (blue) was used to counterstain nuclei. Representative immunofluorescent images were taken at 1000 X magnification (scale bar: 10 µm). (**G**) Immunofluorescent signal was quantified on the inner periphery (nuclear periphery) or interior (scattered) of purified nuclei. Results are shown as the percent mean distribution of signal (% nuclei) in 200 nuclei from each IF analysis. Negative controls are included in Figure S2.

We have previously shown that Ago1 and Ago2 exist in the nuclear fraction of mouse cells [11]. To determine if this feature is conserved in human cells, we examined Ago1 and Ago2 cellular distribution in the nuclear and cytosolic fractions of PC-3 (prostate adenocarcinoma) and RWPE-1 (normal prostatic epithelial) cells by immunoblot analysis. Nuclear distribution of endogenous Ago1 and Ago2 proteins was readily detectable in both cellular compartments (Figure 1A, 1B). Stable overexpression of exogenous HA-tagged Ago1 (HA-Ago1) or Ago2 (HA-Ago2) in PC-3 was also detected in both nuclear and cytosolic fractions (Figure 1C). Immunofluorescence (IF) analysis confirmed that the distribution of Ago1 and Ago2 was evident in both the cytoplasm and nucleus of PC-3 cells expressing HA or GFP-tagged Ago proteins, although signal appeared more prominent in the cytoplasm when observing whole cell distribution (Figure S1).

To determine if nuclear Ago proteins are associated with chromatin, we adopted a fractionation protocol [15] designed to selectively isolate chromatin-bound factors (Figure 1D). Immunoblot analysis revealed that Ago1 and Ago2 were detected in both chromatin fractions (P1 and S2), as well as present in the Triton X-100 soluble fraction (S1) comprising non-chromatin bound cellular proteins such as tubulin (Figure 1E); consistent with the canonical functions of Ago proteins in post-transcriptional gene silencing (PTGS) mechanisms. RNA polymerase II (RNAP II) was also detected and served as a marker for chromatin association (Figure 1E). Taken together, these results suggest that Ago1 and Ago2 are present in the nucleus of human cells in which a subfraction is bound to chromatin.

To analyze Ago protein distribution in only the nuclear compartment, we performed IF on isolated nuclei from the HA-Ago1 and HA-Ago2 stable cell lines. As shown in Figure 1F, 1G, Ago1 signals were generally scattered throughout the nuclear interior, whereas Ago2 was predominantly found on the inner nuclear periphery. Negative controls omitting the primary antibody or using cells without HA tag yielded no staining at all (Figure S2). This data indicates Ago1 and Ago2 have different nuclear localization patterns, which may reflect differences in their nuclear function.

### Nuclear Ago1 interacts with RNA polymerase II

**Figure 2:**
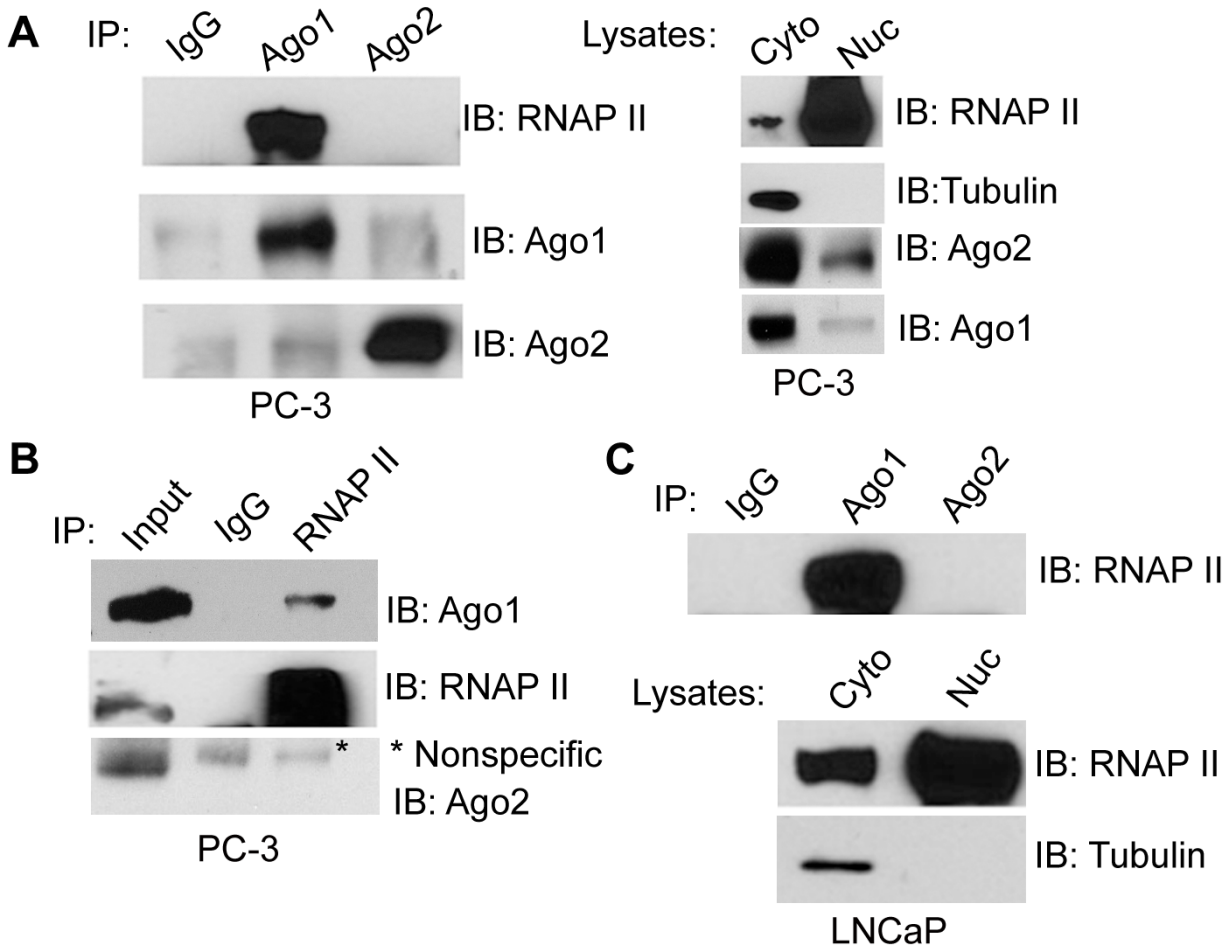
Nuclear Ago1 interacts with RNAP II. (**A**) Immunoprecipitation (IP) assays were performed on nuclear extracts from PC-3 cells using Ago1 or Ago2 antibodies. IgG served as a negative IP control. Immunoprecipitates were analyzed by immunoblotting (IB) with the indicated antibodies. RNAP II and Tubulin were also detected by IB analysis to validate nuclear (Nuc) and cytoplasmic (Cyto) fractions. (**B**) Reciprocal IP analysis was performed on nuclear extracts from PC-3 cells using an antibody specific to RNAP II. IB detected pulldown of Ago1 and RNAP II but not Ago2. Input control represents 10% nuclear extract used for IP. * denotes a nonspecific band. (**C**) IP was performed on nuclear extracts from LNCaP cells as in (A). Nuclear (Nuc) and cytoplasmic (Cyto) fractions were confirmed by IB analysis.

Ago proteins have been implicated in regulating transcriptional mechanisms mediated by small RNA duplexes including gene activation and silencing [11], [16]. To determine if Ago proteins directly interact with transcriptional machinery, we performed immunoprecipitation (IP) assays on nuclear extracts from PC-3 cells using antibodies specific to endogenous Ago1 or Ago2 and immunoblotted for RNAP II. As shown in Figure 2A, RNAP II strongly co-precipitated with Ago1, but not Ago2. We further performed reciprocal RNAP II IP experiments followed by immunoblotting for Agos as well as TFIIB, a known RNAP II interacting protein, as a positive control (Figure S3). The result further confirmed RNAP II association with Ago1, but not Ago2 (Figure 2B). This interaction was also conserved in nuclear extracts from LNCaP (human prostate adenocarcinoma) cells (Figure 2C).

**Figure 3:**
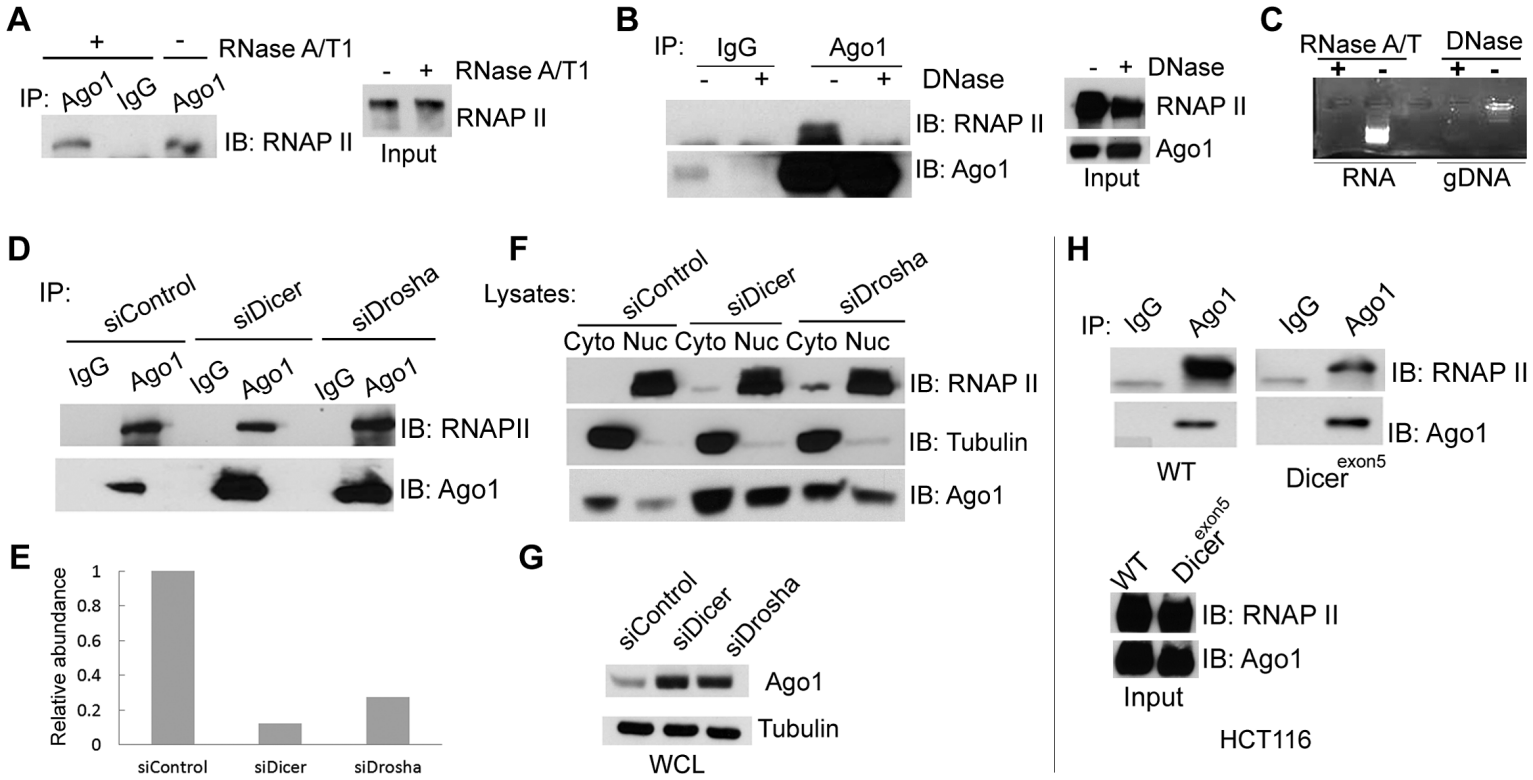
Dependence of nuclear Ago1-RNAP II interaction on RNA, DNA and miRNA biogenesis. (**A and B**) IP was performed on nuclear extracts from PC-3 cells pre-treated with the indicated nuclease treatments. Immunoprecipitates were analyzed by immunoblotting (IB) using RNAP II or Ago1 antibodies. Input represents 10% nuclear extract used for IP (**C**) Total cellular RNA (RNA) and genomic DNA isolated (gDNA) from PC-3 cells were digested with RNase A/T or DNase to confirm the effectiveness of the treatments in (A) and (B).(**D** and **E**) PC-3 cells were transfected with siControl, siDicer, or siDrosha at 50 nM for 72 hrs. IP was performed on nuclear extracts using Ago1 antibody. Immunoprecipitates were analyzed by IB using RNAP II or Ago1 antibodies (D). Densitometry analysis quantified levels of RNAP II and Ago1 pulled down in each IP sample. RNAP II signal was normalized to Ago1 levels to determine the relative ratio of RNAP II bound to nuclear Ago1. The histogram depicts the ratio between RNAP II and Ago1 levels (E). (**F** and **G**) The levels of Ago1 and RNAP II were detected in the cytosolic and nuclear fractions (F) or in whole cell lysate (WCL) (G) by IB following Dicer or Drosha knockdown. RNAP II and tubulin served as nuclear and cytoplasmic markers, respectively. (**H**) IP analysis was performed in HCT116 cells possessing wild-type (WT) or mutant Dicer (Dicer^exon5^) using Ago1 antibody as in (A–F). Input represents 10% nuclear extract used for IP.

To address whether the Ago1-RNAP II interaction requires RNA species as intermediates, nuclear extracts were digested with a cocktail of RNase A and T1 (RNase A/T1) prior to IP (Figure 3A–C). RNase A/T1 treatment did not disrupt interactions between Ago1 and RNAP II (Figure 3A). Although it is possible that RNA molecules may have been protected from digestion by Ago1 or its associated protein complex [17], the data implies Ago1-RNAP II interactions are stable following depletion of nuclear single-stranded RNA species. To determine whether the interactions are DNA dependent, we treated the nuclear extracts with DNase and found that DNase treatment abolished Ago1-RNAP II association (Figure 3B, 3C), suggesting that DNA is required for their interaction.

To test if depletion of miRNA and/or components of the miRNA biogenesis pathway alter the Ago1-RNAP II interaction, we transfected PC-3 cells with siRNA designed to specifically knockdown Dicer (siDicer) or Drosha (siDrosha) (Figure S4A, S4B). Treatment with either siDicer or siDrosha resulted in ≥50% declines in several highly expressed miRNAs implying global downregulation of miRNA maturation (Figure S4C). It should be noted that siDicer and siDrosha treatments also upregulated endogenous protein levels of Ago1 including its nuclear abundance (Figure 3D–G), which may have resulted from a possible compensation mechanism in response to miRNA depletion [18]. Regardless, a moderate decrease in the amount of Ago1-associated RNAP II was observed following Dicer knockdown; the ratio of bound RNAP II to nuclear Ago1 decreased by ∼70% following siDicer treatment (Figure 3E),

Mutation to Dicer at exon 5 has been used to generate a stable cell line (Dicer^exon5^) derived from HCT116 (colorectal carcinoma) cells with impaired helicase function that interferes with miRNA maturation [19]. IP experiments revealed that co-immunoprecipitation of RNAP II with Ago1 antibody was reduced in Dicer^exon5^ cells compared to wild-type (WT) controls (Figure 3H), although the protein levels of neither Ago1 nor RNAP II changed in Dicer knockout line compared to its parental cells (Figure 3H). Taken together, these results indicate that Ago1 directly interacts with the core transcription machinery in human cells, which may require Dicer activity and/or the miRNA species it processes.

### Genome-wide mapping of Ago1 binding sites

The physical association between Ago1 and RNAP II strongly suggests that Ago proteins may participate in transcriptional gene regulation by interacting with chromatin. Previous studies have demonstrated that Ago proteins programmed with small RNAs can bind to gene bodies or promoters by using chromatin IP (ChIP) assays [11], [12], [20]. To provide a more global view of nuclear Ago interactions, we mapped Ago1 and Ago2 binding in the genome by ChIP coupled with massively parallel sequencing (ChIP-seq). Antibody validation confirmed that ChIP antibodies for Ago1 and Ago2 had no detectable cross-reactivity ([21] and Figure S5A–C, Figure 2A) and are highly specific for RNA-protein IP and ChIP based applications ([20] and Figure S5D). ChIP-seq was also performed for H3K4me3; a histone mark associated with active gene transcription [22]. DNA quality and fragment size distribution for each library was roughly equivalent (Figure S6A). Approximately 80–100 million sequencing reads were obtained from each ChIP-seq library of which ∼80–90% could be uniquely mapped back to the human genome (Table S1, S2). To identify Ago1, Ago2, and H3K4me3-enriched regions, we applied the CCAT (control-based ChIP-seq analysis tool) peak calling algorithm [23] to the raw reads and obtained 110,533 Ago1, 144 Ago2, and 16,729 H3K4me3 peaks (Table S2). By conservatively setting the false discovery rate (FDR) cutoff to 0.054 based on independent ChIP validation results (Figure S6B–D), we obtained 44,684 Ago1 and 16,151 H3K4me3 bona fide peaks (Table S2, S3, S4). None of the Ago2 peaks passed the FDR cutoff (Table S2); therefore, we focused our subsequent analyses only on Ago1.

**Figure 4:**
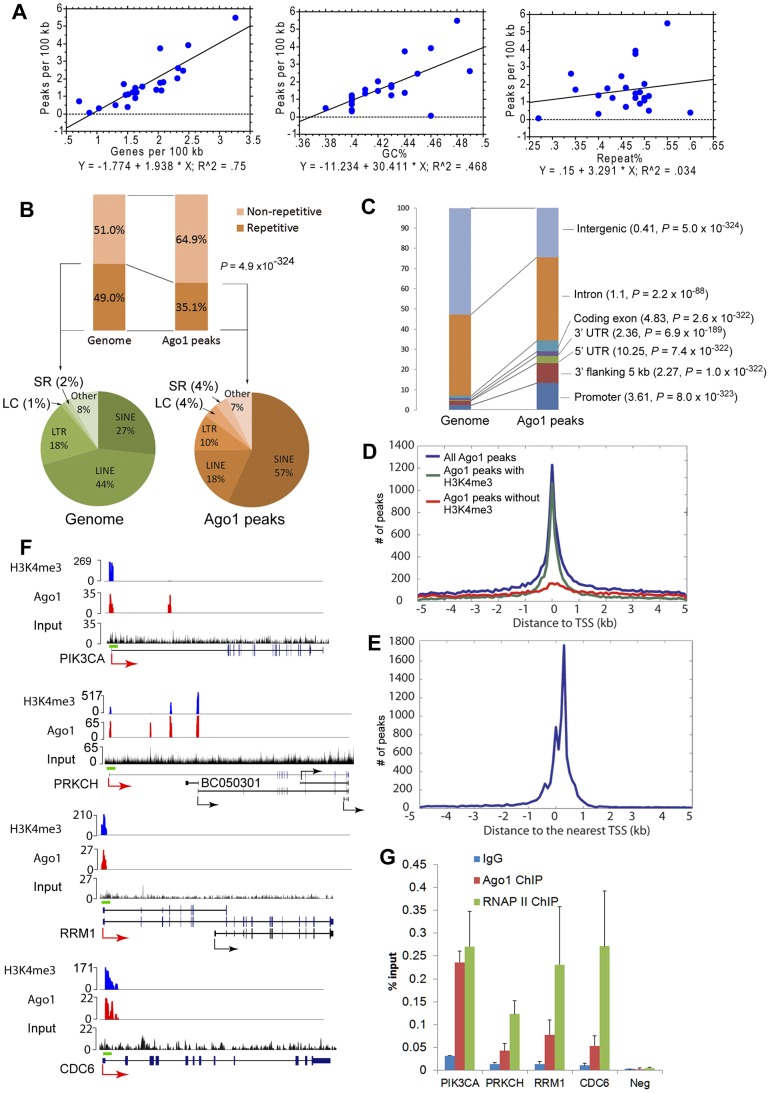
Ago1 is preferentially bound to euchromatic regions on chromosomes. (**A**) Shown are bivariate scattergrams with regression analysis between Ago1 peak density (peaks/100 kb) and gene density (genes/100 kb, upper panel), GC% (middle panel), or Repeat% (lower panel). (**B**) Relative density of repetitive and non-repetitive sequences present in the Ago1-bound sequences. The percentages define Ago1-bound sequence composition in comparison to abundance in human genome. Repetitive sequence composition is subdivided in the pie charts to include low complexity (LC), simple repeats (SR), long terminal repeats (LTR), short interspersed elements (SINE), and long interspersed elements (LINE). (**C**) Relative density of Ago1 peaks located in genic (i.e. promoter, 5′UTR, coding exon, intron, 3′UTR, and 3′ flanking region) and intergenic regions. The numbers in the parentheses indicate enrichment ratio relative to the genome with statistical analysis. (**D**) Distribution of Ago1 peaks relative to TSSs of annotated genes and their correlation with H3K4me3 peaks. (**E**) Distribution of H3K4me3 peaks relative to TSSs. (**F**) Genome browser views of Ago1 (red) and H3K4me3 (blue) peaks on 4 representative AbGs including PIK3CA, PRKCH, CDC6 and RRM1. Y-axis is normalized number of reads. All peaks passed the FDR cutoff are shown. Input tracks are included as controls. Major transcription start site (TSS) for each gene is shown by the red arrow. Alternative TSSs are denoted by black arrows. Green bars above gene structures correspond to ChIP amplicons used below. (**G**) Independent ChIP analyses of the representative promoters were performed in PC-3 cells. Ago1 and RNAP II occupancy were determined by qPCR using primer sets encompassing the green bars designated in (F). Results are shown as mean % input ± SD from 3 independent experiments. IgG was used as a negative control. Neg: negative control region.

On average, Ago1 peaks were found once in every 70 kb of genomic sequence (Table S5) having a typical size of ∼1 kb, while the size of H3K4me3 peaks were generally broader (Figure S6E, S6F). Ago1 peaks were neither evenly distributed on chromosomes nor on genes; rather, their distribution on chromosomes correlated strongly with gene density (R^2^ = 0.75, *P*<0.0001) and GC% (R^2^ = 0.468, *P* = 0.0001), but not with % repetitive sequences (R^2^ = 0.034) (Figure 4A and Table S5). For example, the highest Ago1 binding density was seen on gene-dense chromosomes 19 and 17, while lowest Ago1 binding was on chromosomes Y and 13, which have the lowest gene density (Figure 4A, Figure S7, and Table S5). When multiple regression analysis was applied, gene density becomes the sole determinant of Ago1 binding density on chromosomes (*P*<0.001, Table S6). In addition, the majority of Ago1 peaks do not overlap chromosomal “HOT” (high occupancy transcription-related factors binding) regions [24], suggesting that Ago1 peaks we identified are not due to experimental or computational artifacts (Text S1).

Overall, Ago1-bound sequences were largely (64.9%) non-repetitive (Figure 4B). Statistical analysis indicated that Ago1 is associated with significantly less (35.1%) repetitive elements compared to overall abundance in the genome (49%, *P* = 4.9×10^−324^) (Figure 4B). Nonetheless, the major fraction of bound repetitive sequence consisted primarily of SINE, LINE, and LTR transposable elements (Figure 4B). SINE (56.8%), low complexity (4.1%) and simple repeat (3.6%) elements were overrepresented compared to their respective frequency in the genome, while LINE (18.2%) and LTR (10.2%) repeats were depleted in Ago1-bound sequences (Figure 4B). Nuclear RNAi has been implicated in transposon regulation in yeast and other eukaryotes by interacting with noncoding transcripts generated from repetitive sequence [25]. It is possible that transposable elements also mediated Ago1 interactions in the nucleus of human cells by a similar manner.

### Ago1 is pervasively associated with promoters of actively transcribed genes

Ago1 peaks were also categorized based on gene proximity to include intragenic regions (i.e. introns, exons, and UTRs) and adjacent sequences (i.e. promoters and 3′ flanking region) within 5 kb of gene bodies. Overall, a majority of the reads corresponded to these genic locations. Compared to their respective composition in the genome, all genic regions were overrepresented in the Ago1 library including promoters, 5′UTRs, exons, introns, 3′UTRs, and 3′ flanking regions by 3.61-, 10.25-, 4.83-, 1.1-, 2.36-, and 2.27-fold, respectively (Figure 4C). In contrast, Ago1 peaks were significantly underrepresented in intergenic regions (0.41-fold, *P* = 5×10^−324^) (Figure 4C).

Given that Ago1 binding was primarily genic, we evaluated Ago1 peak distribution within ±5 kb of transcription start sites (TSS) of annotated genes. We found that the majority of Ago1 peaks mapped to a region within ±1 kb of TSSs in a distribution pattern similar to H3K4me3 peaks (Figure 4D, 4E). In fact, by further stratifying Ago1-bound genes (AbGs) for the presence or absence of H3K4me3 at TSSs, we found that within the ±1 kb region, 65.2% of Ago1 peaks overlapped with the H3K4me3 mark (Figure 4D). This data implies that Ago1 pervasively associates with chromatin at TSSs of transcriptionally active genes.

### Ago1 binding events correlate with active gene regulation

Select examples of AbGs include PIK3CA, PRKCH, CDC6, and RRM1, which have overlapping Ago1 and H3K4me3 peaks proximal to their TSSs (Figure 4F). To determine if RNAP II was also bound to AbGs, we performed ChIP analysis at the promoters of each gene. As shown in Figure 4G, we detected an enrichment of RNAP II as well as Ago1 at each TSS. Collectively, these results indicate Ago1, H3K4me3, and RNAP II are present at the promoters of the example AbGs.

**Figure 5:**
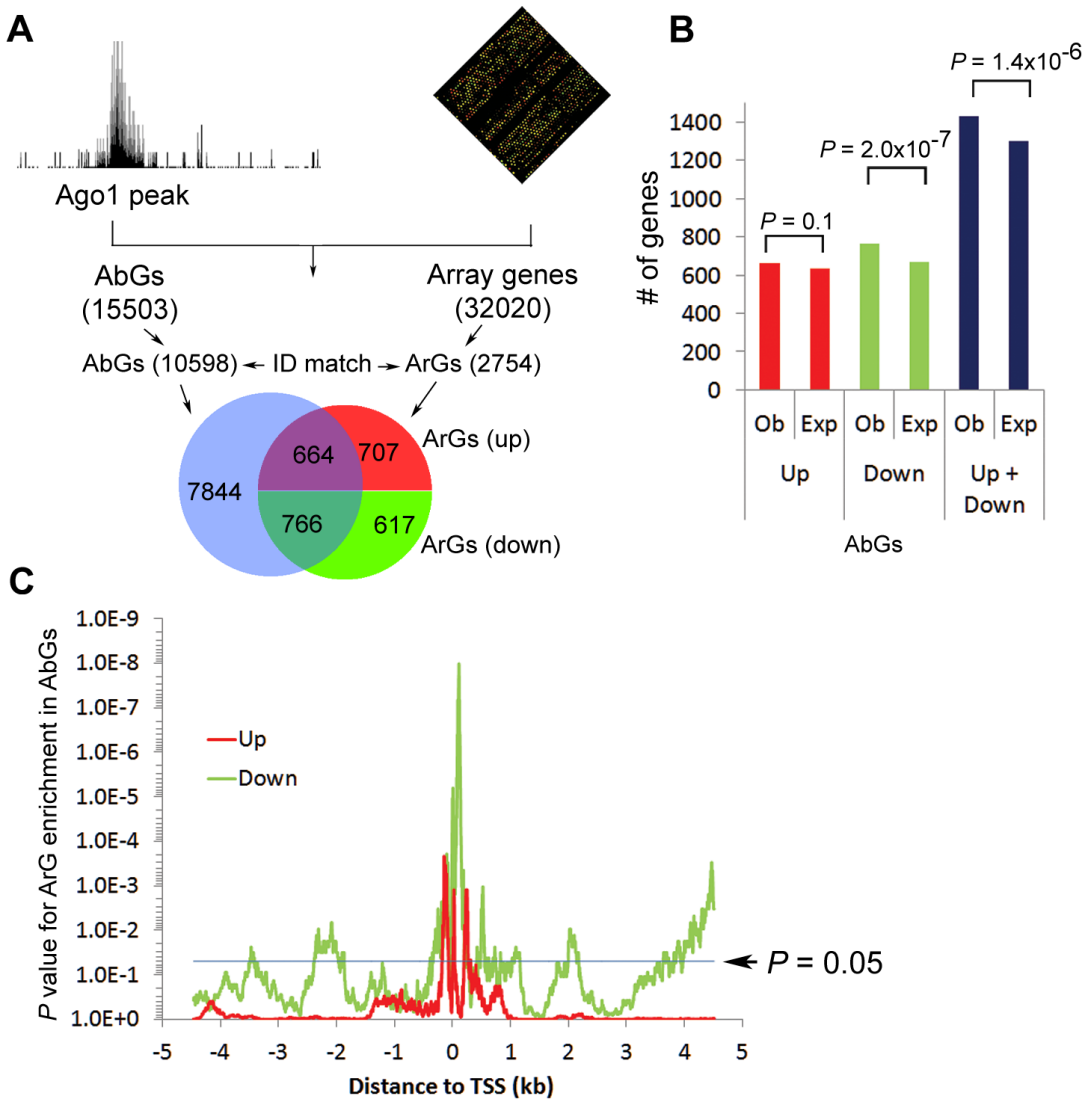
Combined ChIP-seq and microarray analysis reveals that Ago1 depletion affects expression of Ago1-bound genes. (**A**) Schematic of the correlation analysis between AbGs and ArGs. The Venn diagram shows overlap between AbGs and ArGs. Only genes with a matched Ensembl gene ID were included resulting in 10598 AbGs and 2754 ArGs. Red and green groupings correspond to up- (Up) and downregulated (Down) ArGs as a result of Ago1 knockdown. (**B**) The total number of experimentally observed (Ob) up- and downregulated ArGs within the AbG group were compared to the total number of expected (Exp) AbGs predicted to be regulated by Ago1 depletion. (**C**) All *p*-values for up- (red line) and downregulated (green line) ArGs within the AbG set were plotted against the corresponding distance from their Ago1 peak to TSS. Statistical significance is increased for ArGs the closer Ago1 peaks are to TSSs (0). The blue line delineates an arbitrary statistical threshold of *P* = 0.05.

To evaluate the impact of Ago1 perturbation on AbG expression, we performed microarray analysis in PC-3 cells following Ago1 knockdown with a pool of 3 Ago1-specific siRNAs (siAgo1) (Figure S8A). We identified a total of 3156 Ago1-responsive genes (ArGs) including 1592 up- and 1564 downregulated genes defined by >1.2-fold change in expression with a *P* value<0.05 (Table S7, S8 and Figure S8B). Twenty three genes were selected and independently assessed by qRT-PCR to confirm changes in gene expression (Figure S8C, 8D). AbGs identified by ChIP-seq analysis were subsequently correlated to the changes in global gene expression following Ago1 depletion (Figure 5A). The results indicated that 48.3% of up- and 55.4% of downregulated genes were also bound by Ago1 within 5 kb of their TSSs (Figure 5A) and the overlap between AbGs and ArGs are significantly higher than expected by chance (*P* = 1.4×10^−6^, Figure 5B, blue bars). However, when we stratified ArGs by up and downregulation, correlation was statistically significant only for downregulated ArGs (*P* = 2.0×10^−7^, Figure 5B, green bars) and not upregulated ArGs (red bars, *P* = 0.1, Figure 5B, red bars), suggesting that AbGs are more likely to be downregulated when Ago1 is perturbed.

Furthermore, we examined the positional effect of Ago1 binding (within ±5 kb distance) on changes in gene expression in response to Ago1 perturbation. To this end, we calculated the correlation between changes in gene expression and Ago1 binding events on the same gene for each location within −5 kb∼+5 kb region shifting one basepair each time. In consistent with the overall correlation analysis (Figure 5B), Ago1 binding events have a better correlation with down- (Figure 5C, green line) than upregulated (Figure 5C, red line) ArGs. The closer Ago1 binding was to the proximal promoter region, the greater the statistical significance was for enrichment of ArGs, especially for downregulated ArGs, with the enrichment for downregulated ArGs peaked at +111 location (*P* = 1.0×10^−8^) and upregulated ArGs at −135 location (*P* = 0.002) (Figure 5C). Taken together, the correlation between AbGs and downregulated ArGs suggests that Ago1 plays a positive role in maintaining transcription of a subset of genes. It is important to note that our data does not rule out the possibility Ago1 may also be functioning to suppress gene expression through promoter interactions for certain genes.

### Ago1-bound sequences contain putative miRNA target sites

**Figure 6:**
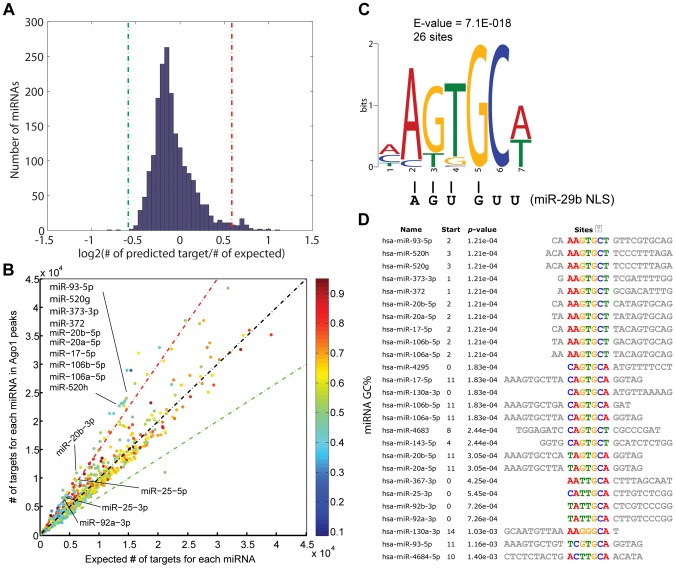
Ago1-bound sequences contain potential miRNA target sites. (**A**) Distribution of ratios between the number of predicted target sites in Ago1 peaks vs. control sequences of the same lengths for all known human miRNAs. The red and green lines represent 1.5-fold enrichment and depletion cutoffs, respectively. (**B**) Scatter plot comparing the number of predicted miRNA target sites in Ago1-bound peaks vs. control sequences. Each dot represents one human miRNA. Dot color denotes miRNA GC% content. Color scale is located to the right of the plot. The red and green lines represent 1.5-fold enrichment and depletion cutoffs, respectively. Indicated are select oncomiRs from the miR-17-92 and miR-106b-25 clusters and miR-520/373 family, which all have enriched putative target sites in Ago1-bound sequences. (**C**) Sequence analysis of all 49 miRNAs enriched for target sites within the Ago1-bound peaks identified a common motif (AGUGCA/U), which resembles the nucleic acid-based NLS of miR-29b (AGUGUU). (**D**) The motif appears 26 times in 19 out of the 49 enriched miRNAs.

miRNAs have been shown to regulate gene transcription by binding to promoter sequences in an Ago-dependent manner [7], [11], [16], [26]. Since Ago proteins do not possess a known DNA binding domain based on protein sequence and structural analysis [27], [28], Ago1-chromosomal interactions might be mediated by miRNAs. As such, we performed miRNA target prediction analysis on the Ago1-bound DNA sequences identified by ChIP-seq. Compared to random selected matched control sequences, the frequency of putative target sites in Ago1-bound peaks were roughly equivalent for most miRNAs (Figure 6A, 6B). However, a total of 49 miRNAs were found to have a statistically higher number of target sites in the Ago1-bound peaks compared to the control sequences (>1.5 fold enrichment, *P* = 0∼6×10^−41^), while only 3 miRNAs, function of which is unknown, have higher number of targets in the control sequences (Figure 6A, 6B and Table S9). Interestingly, approximately one third of the 49 miRNAs are known oncomiRs including those from the miR-17-92 and miR-106b-25 clusters, as well as the miR-520/373 family (Figure 6B, Table S9).

We also preformed motif analysis on each miRNA with enriched target sites in Ago1-bound sequences. A common motif “AGUGCU/A” was found in 19 out the 49 miRNAs; 7 of which contained two incidences of this motif (Figure 6C, 6D). Interestingly, a similar motif (AGUGUU) was identified in the 3′terminus of miR-29b, which functions as a nucleic acid-based nuclear localization signal (NLS) [29]. Although the significance of our motif in context to Ago1-bound sequences is unknown, it shares ∼83% homology with the miR-29b NLS (Figure 6C). As certain miRNAs are known to preferably accumulate in the nucleus [30], the identification of putative target sites at Ago1-bound peaks supports the idea that such miRNAs may play a role in directing Ago1-chromomal interactions.

### Ago1 contributes to active gene regulation

**Figure 7:**
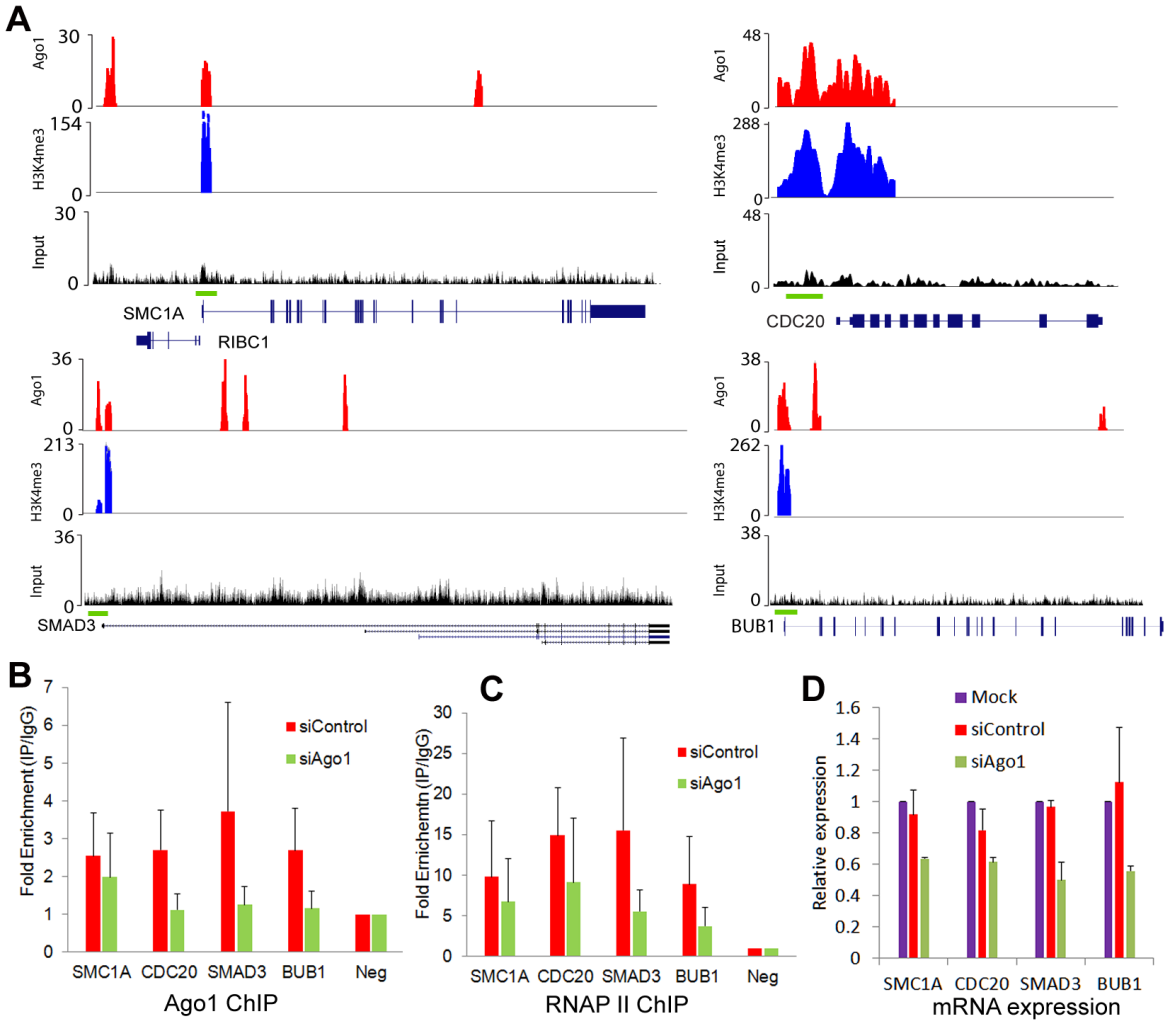
Ago1 knockdown reduces occupancy of Ago1 and RNAP II at gene promoters and downregulates gene expression. (**A**) Genome browser views of Ago1 (red) and H3K4me3 (blue) peaks on 4 representative AbGs including SMC1A, CDC20, SMAD3, and BUB1. Y-axis is normalized number of reads. All peaks passed the FDR cutoff are shown. Input tracks are included as controls. Green bars above gene structures correspond to ChIP amplicons used below. (**B** and **C**) PC-3 cells were transfected with siControl or siAgo1 for 48 hrs. ChIP analysis was performed to determine Ago1 and RNAP II occupancy at the representative promoters using qPCR in conjunction with the primer sets encompassing the green bars designated in (A). Results are shown as mean fold enrichment relative to negative control region (Neg) ± SD from 3 independent experiments. IgG served as a negative control. (**D**) mRNA expression levels of SMC1A, CDC20, SMAD3 and BUB1 were quantified by qPCR (mean ± SD from 3 independent experiments). Values were normalized to GAPDH.

**Figure 8:**
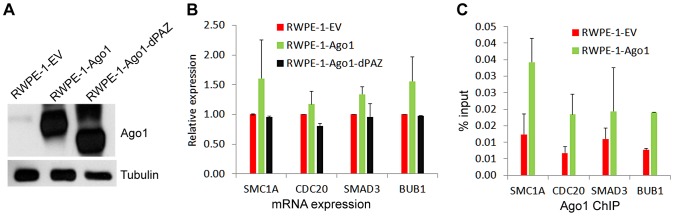
Overexpression of Ago1 leads to increase Ago1 binding of promoters and increased expression of their associated genes. (**A**) Immunoblot analysis confirms stable overexpression of Ago1 or the PAZ deletion mutant (Ago1 dPAZ) in RWPE-1 cells. Transduction with viral particles generated from an empty vector (EV) was used to establish a stable control cell line. Note the reduction in molecular weight of Ago1 dPAZ protein as a result of domain deletion. Detection of tubulin served as a loading control. (**B**) Relative mRNA expression levels of the indicated genes were quantified by qPCR (mean ± SD from 2 independent experiments) in each stable RWPE-1 cell lines. Data was normalized to GAPDH. (**C**) ChIP analysis was performed to determine Ago1 occupancy at the promoters of the indicated genes using qPCR in conjunction with the primer sets encompassing the green bars designated in Figure 7A. Results are shown as mean % input ± SD from 2 independent experiments. IgG served as a negative control.

To test the regulatory effect of Ago1 binding on gene promoters, we depleted Ago1 in PC-3 cells using siAgo1 and evaluated its effect on 4 ArGs (i.e. SMC1A, CDC20, SMAD3 and BUB1) with overlapping Ago1 and H3K4me3 peaks at their TSSs (Figure 7A). ChIP analysis revealed reductions in bound Ago1 at the promoters for each gene (Figure 7B). Moreover, knockdown of Ago1 reduced RNAP II occupancy at TSSs (Figure 7C) with corresponding decreases in gene expression levels (Figure 7D). We also generated stable cell lines in RWPE-1 (non-malignant prostate epithelium) cells overexpressing Ago1 or a deletion mutant lacking the PAZ domain (Ago1 dPAZ) (Figure 8A), which is known to interfere with efficient miRNA loading into Ago proteins [31]. Ago1 overexpression resulted in a moderate induction of each gene (Figure 8B), while PAZ deletion attenuated this response (Figure 8B), further supporting a role for miRNAs in directing Ago1-chromosomal interactions. Furthermore, we performed Ago1 ChIP for the 4 example genes and were able to detect in Ago1 overexpressing RWPE-1 cells a concurrent increase in Ago1 binding at the same sites near TSSs detected in PC-3 cells (Figure 8C). Collectively, these results suggest Ago1 contributes to positive gene regulation of select ArGs by interacting with gene promoters and stimulating RNAP II enrichment.

### Ago1-bound genes are enriched for cancer-related pathways

Three overlapping AbG sets were defined to include AbGs-5 kb, -1 kb and -0.5 kb, which consist of genes with at least one Ago1 peak within ±5, ±1, and ±0.5 kb away from TSSs, respectively. AbGs-5 kb, -1 kb and -0.5 kb respectively contain 15503, 10074, and 8057 unique genes encompassing 27.5%, 17.9%, and 14.3% of all annotated genes in Ensembl human genome database (Table S10, S11, S12). Interestingly, clustering AbGs-5 kb, -1 kb or -0.5 kb genes by their chromosomal location reveal several cytobands implicated in different human cancers that are highly overrepresented (Figure S9, Table S13, S14). For example, the top-enriched cytobands 19p13.3 and 16p13.3 have been established by numerous studies to be susceptibility loci for several types of cancers including prostate, breast, thyroid, and lymphoma [32]–[35].

**Figure 9:**
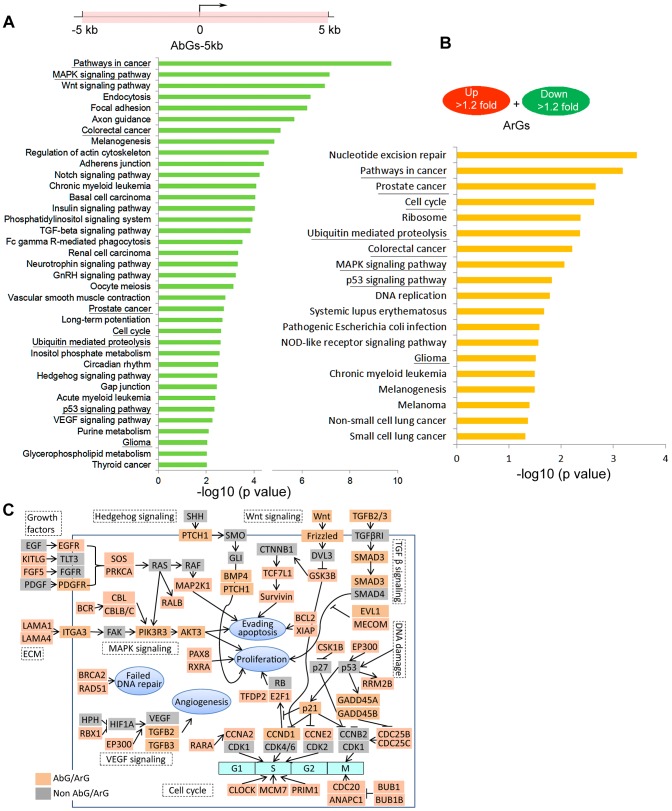
Ago1-bound and Ago1-responsive genes are enriched for cancer pathways. (**A**) Illustrated is a schematic representation defining the AbGs-5 kb gene set as possessing Ago1 peaks within ±5 kb of their TSSs (marked in pink). Indicated are the top enriched pathways (*P*<0.01) for all AbGs-5 kb genes. KEGG pathway enrichment analysis was performed by using the DAVID bioinformatics tool. (**B**) Indicated are the top enriched pathways (*P*<0.05) for all up- and downregulated ArGs as determined by KEGG pathway enrichment analysis using the DAVID tool. All underlined pathways are shared by the AbGs-5 kb and ArG gene sets. (**C**) The schematic illustrates exemplary AbGs and ArGs involved in oncogenic pathways. Major gene nodes for the indicated pathways are represented by genes grouped as Ago1-bound/regulated genes (AbGs/ArGs; pink) or non-Ago1 bound/regulated genes (non-AbGs/ArGs; grey).

Gene pathway enrichment analysis further revealed a number of oncogenic pathways overrepresented by AbGs. The top 5 KEGG pathways highly enriched in AbGs-5 kb genes include “pathways in cancer” (*P* = 1.9×10^−10^), “MAPK signaling” (*P* = 1.7×10^−8^), “Wnt signing” (*P* = 1.1×10^−7^), “endocytosis” (*P* = 4.3×10^−7^), and “focal adhesion” (*P* = 6×10^−7^) (Figure 9A). Many proto-oncogenes and proliferation-promoting genes are exemplified in these pathways including growth factors, tyrosine/serine/threonine kinases, G-protein coupled receptors, membrane-associated G-proteins, and nuclear DNA-binding/transcription factors (Table S15). These enrichments hold when AbGs are narrowed down to AbG-1 kb and AbG-0.5 kb genes (Figure S10A, S10B). For instance, SMC1A, CDC20, SMAD3 and BUB1 are all example AbG-0.5 kb genes known to promote cell cycle progression and proliferation in various cancer cell types [36]–[39]. Gene Ontology (GO) classification of AbGs-5 kb genes also show enrichment for gene categories that regulate metabolic processes, transcription, cell cycle, chromatin modification, and cell death (Figure S10C).

KEGG pathway enrichment analysis also revealed that ArGs shared several cancer-related pathways with AbGs-5 kb genes including “MAPK signaling”, “p53 signaling”, “cell cycle”, “prostate cancer”, “colorectal cancer”, etc. (Figure 9B). Further GO analysis revealed that up- and downregulated ArGs were enriched in distinct biological processes with the latter significantly overrepresented by processes important for cancer growth/development including cell cycle, mitosis, DNA repair, chromosome organization, etc. (Figure S11A, S11B).

Analysis of Ago1 protein levels in non-tumorigenic (RWPE-1 and PWR-1E) and cancerous (PC-3, DU145, LNCaP, RV1, CWR22R, and C4-2) prostate cell lines indicated Ago1 is generally expressed at significantly higher levels in cancer cell lines (Figure S12A). Furthermore, knockdown of Ago1 in PC-3 cells caused G0/G1 arrest as indicated by the increase in G0/G1 cell number and corresponding reductions in S and G2/M populations (Figure S12B, S12C). Our data suggests Ago1 may be involved in oncogenic processes, in part, through its nuclear activity by affecting the expression of genes involved in cell growth/survival. In support, integrated analysis of ChIP-seq and gene expression profiling places Ago1 in various major cancer-related signaling pathways involved in regulating DNA damage response, mitogenic signaling, cell cycle, angiogenesis, and apoptosis (Figure 9C).

## Discussion

It has become clear that Ago proteins participate in gene regulation at multiple levels. In the present study, we reveal another layer to Ago1 in regulating gene expression within the nucleus of human cancer cells. We provide biochemical evidence that nuclear Ago1, but not Ago2, directly associates with RNAP II. ChIP-seq analysis indicates Ago1 is pervasively bound to multiple genomic loci including repetitive elements of transposons and euchromatic sites as defined by the histone mark H3K4me3. Interestingly, this observation is consistent with the chromosomal binding profiles of drosophila Ago2 (dAGO2); the primary Argonaute for mediating RNAi and miRNA function in the fly [15], [40]. Additionally, Ago1 binding at gene promoters functionally impacts active gene transcription as its loss of function results in reduced Ago1 and RNAP II occupancy at TSSs with corresponding reductions in gene expression, whereas gain of function causes the opposite changes. Our data represents the first landscape of Ago1-chromosomal interactions in human cancer cells, while revealing a novel non-canonical function for Ago1 in regulating gene expression.

It is currently unclear how Ago1 is targeted to selected chromosomal loci. Our analyses imply that miRNA may be involved in mediating interactions between nuclear Ago1, chromatin, and/or RNAP II. Ago1-bound sequences contained putative miRNA target sites and its binding activity to RNAP II was suppressed by perturbing Dicer function; an essential protein involved in miRNA maturation. Additionally, deletion of the RNA-binding domain (PAZ) in Ago1 interfered with gene activation further implicating a role for RNA (i.e. miRNA) in this process. In support, it has been reported that transfection of exogenous miRNA can promote enrichment of Ago proteins at highly-complementary sites in gene promoters to manipulate transcription [7], [11], [16]. Depletion of nuclear single-stranded RNAs by RNase A/T1 did not interfere with Ago1-RNAP II association; however, Ago1 may be loaded with miRNA forming a duplex with complementary target sequence and protecting bound RNA from RNase A/T1 digestion in manner similar to canonical target recognition [17], [41]. As we have not definitely confirmed the presence of miRNAs in these nuclear Ago1 complexes, it is also possible other classes of small RNA species mediate Ago1 interactions with chromatin. For instance, recent deep sequencing studies have shown that Ago1 can associate with small RNA species from non-miRNA sources [42], [43].

In contrast to Ago1, Ago2 apparently lacked pervasive association with chromatin. Additionally, it did not immunoprecipitate with basal transcription machinery (i.e. RNAP II). Although we cannot absolutely rule out technical reasons for the lack of Ago2 binding, the difference in binding may be reflective of their differential nuclear distribution as revealed by IF microscopy (Figure 1E). Ago1 and Ago2 have been reported to exhibit intrinsic preferences when selecting and/or loading RNA molecules. For instance, studies have shown that Ago2 binds perfect-complementary RNA duplexes (e.g. siRNAs) with higher affinity than Ago1; whereas, Ago1 preferably associates with duplexes containing bulges and mismatched bases (e.g. miRNA) [44], [45]. This intrinsic segregation in RNA binding may also be a key determinant in mediating Ago interactions in the nucleus. Alternatively, nuclear Ago2 may be sequestered to the nuclear envelope and only associate with chromatin in a signal-dependent manner. In support, cellular senescence has been shown to trigger nuclear accumulation of Ago2 and binding at gene promoters [8].

It is noteworthy that the magnitudes of gene expression changes for a vast majority of genes in response to Ago1 perturbation were less than two-fold. This observation is consistent with post-transcriptional gene regulation by miRNA [46] and suggests that the role of Ago1 is fine-tuning gene expression in a miRNA dependent manner both at the transcriptional and post-transcriptional levels. The short term (48 hrs) transfection of Ago1 siRNA may also be accounted for the subtle changes in gene expression. We chose this duration to minimize detecting potential secondary regulation but at the same time we might have missed the maximum responsiveness of gene expression to Ago1 perturbation.

In the cytoplasm, Ago proteins elicit pleiotropic effects on gene expression by utilizing miRNA to silence multiple transcripts and regulate various cellular processes [1], [2]. Similarly, nuclear Ago1 also possesses pleiotropy by affecting transcription of multiple genes. In PC-3 cells, Ago1 appeared to preferably drive the expression of genes involved in oncogenic pathways suggesting it may play a role in the cancer phenotype. In support, knockdown of Ago1 by siRNA inhibited cell cycle progression. However, its effects on cancer may be context dependent and vary between different cell types based on both its cytosolic and nuclear activities, as well as the gene profile it regulates. It would be of future interests to understand the crosstalk between Ago-mediated gene regulatory networks and oncogenic signaling pathways.

## Materials and Methods

### Cell culture

PC-3, LNCaP, DU145, LAPC4, RV1, CWR22R, C4-2, and HCT116 cell lines (ATCC) were maintained in RPMI-1640 media (UCSF Cell Culture Core) supplemented with 10% fetal bovine serum (Hyclone), penicillin G (100 U/mL), streptomycin (100 µg/mL) in a humidified atmosphere of 5% CO_2_ at 37°C. RWPE-1 and PWR-1E cells were cultured in serum-free keratinocyte medium supplemented with 5 ng/ml human recombinant epidermal growth factor and 0.05 mg/ml bovine pituitary extract.

### Plasmids and gene overexpression

Vectors pIRESneo-FLAG/HA-Ago1 (Addgene #10820) and pIRESneo-FLAG/HA-Ago2 (Addgene #10822) were used to establish stable cell lines overexpressing HA-tagged Ago1 (PC3-HA-Ago1) and Ago2 (PC3-HA-Ago2), respectively. Briefly, PC-3 cells were transfected with each corresponding vector and single colonies were subcultured following selection with G418. GFP-Ago1 (Addgene #21534) and GFP-Ago2 (Addgene #11590) plasmids were transiently transfected into PC-3 cells and imaged by fluorescence microscopy. Full-length human Ago1 and the PAZ deletion mutant (Ago1 dPAZ) were amplified from pIRESneo-FLAG/HA-Ago1 and pIRESneo-FLAG/HA-Ago1dPAZ, respectively. Each amplicon was cloned into the lentiviral cDNA expression vector pCDH-EF1-MCS-T2A-copGFP (System Biosciences) via EcoRI and BamHI restriction sites. For lentivirus mediated overexpression, lentivirus particles were generated by the ViraPower Lentiviral Expression System (Invitrogen) and used to infect RWPE-1 cells to generate stable cell lines. Expression of all constructs was confirmed by immunoblot analysis.

### Immunofluorescence microscopy

PC-3 cells were seeded on coverslips at 50% confluency. The following day, cells were washed with PBS and fixed in 4% paraformaldehyde at room temperature for 15 min. Cells were permeabilized in PBS containing 0.3% Triton-X-100 for 10 min, rinsed with PBS, and blocked with 10% goat serum at room temperature for 1 hr. Coverslips were incubated with primary antibodies anti-HA (Cell Signaling,cat # 2367, 1∶200) or anti-Ago2 (Wako, cat # 011-22033, 1∶200) diluted in 10% goat serum at room temperature for 1 hr. Cells were washed with PBS and subsequently treated with anti-mouse FITC antibody (Vector Lab; 1∶200) at room temperature for 1 hr. Coverslips were washed and mounted with mounting media containing DAPI. IF images were captured using a Zeiss AxioImager M1 fluorescence microscope. Purified nuclei for IF analysis were isolated as previously described [47]. Nuclei were fixed on slides with fixative reagent (methanol: acetic acid, v/v 3∶1) at room temperature for 5 min and washed with 4×SSC containing 0.1% Tween 20. The slides were subsequently incubated with anti-Ago1 (Santa Cruz Biotechnology, cat #sc-32657, 1∶200), anti-Ago2 (Wako, cat #011-22033, 1∶200), or anti-HA (Cell Signaling, cat #2367, 1∶200) diluted in dilution buffer (1% bovine serum albumin, 4×SSC, and 0.1% Tween 20) at 4°C overnight. Nuclei were washed and incubated with the appropriate Alexa Fluor® 488 secondary antibodies (Molecular Probes; 1∶200) for 30 minutes at 37°C. Following a series of washes, slides were mounted with DAPI II (Abbot Molecular) and IF signals were analyzed using the CytoVision imaging system (Applied Imaging).

### Chromatin and subcellular fractionation

Chromatin fractionation was performed as previously described [15]. Cell pellets were collected from two 150 mm plates and washed with PBS. Approximately 1/10^th^ of the cell pellet was resuspended in RIPA buffer (50 mM Tris, pH 7.4, 150 mM NaCl, 1% Triton ×-100, 0.5% deoxycholate, 0.1% SDS, protease inhibitor cocktail, and phosphatase inhibitor) and incubated on ice for 30 min to generate whole cell lysate. The remaining pellet was lysed in cold CSKI buffer [10 mM PIPES, pH 6.8, 100 mM NaCl, 1 mM EDTA, 300 mM sucrose, 1 mM MgCl_2_, 1 mM DTT, 0.5% (v/v) Triton X-100, and protease inhibitor cocktail (Roche)]. The lysate was divided into two equal portions and centrifuged at 500×g for 3 min at 4°C. The resulting supernatant was collect and referred to as the S1 fraction. One pellet was washed twice in CSKI buffer and resuspended in RIPA buffer to generate the P1 fraction. The other pellet was resuspended in CSKII buffer [10 mM PIPES, pH 6.8, 50 mM NaCl, 300 mM sucrose, 6 mM MgCl_2_, 1 mM DTT, and protease inhibitor cocktail (Roche)] and treated with DNase (Qiagen) for 30 min. The resulting sample was extracted with 250 mM NH_2_SO_4_ for 10 min at room temperature and centrifuged at 1200×g for 6 min at 4°C to generate the S2 (supernatant) and P2 fractions (pellet). The P2 fraction was subsequently resuspended in RIPA buffer. Cytoplasmic and nuclear fractions were prepared by using the NE-PER Nuclear and Cytoplasmic Extraction Reagents (Thermo Scientific). Whole cell lysate was obtained by lysing cells in RIPA buffer for 15 minutes at 4°C. Lysates were clarified by centrifugation for 15 minutes at 14,000 rpm and supernatants were collected. 30 µg of protein from all fractions was analyzed by immunoblot analysis.

### Immunoprecipitation

Immunoprecipitation was performed according to Cernilogar *et al.* [15]. Approximately 400–800 µg of protein from nuclear extracts was mixed with equal volumes immunoprecipitation buffer [10 mM Tris-HCl, pH 8.0, 150 mM NaCl, 1 mM EDTA, 1 mM DTT, 0.1% NP-40, and protease inhibitor cocktail (Roche)]. In Figure 3A–3C, nuclear extract was treated with 2.5 ul of RNase A/T (Ambion) cocktail for 30 min at 25°C or 100 ng/uL of DNAse I (Roche) for 20 min at 37°C. Each sample was subsequently treated with 5 µg of antibody and incubated overnight at 4°C. Antibody treatments included anti-Ago1 (Wako, clone 2A7, cat# 015-22411), anti-Ago2 (Wako, clone 4G8, cat# 011-22033), anti-RNAP II (Millipore, cat# 05-623), or mouse IgG (Millipore, cat# 12-371). The following day, 40 µl protein G dynabeads were added to each sample and rotated for 2 hrs at 4°C. The beads were subsequently washed five times with 500 µl immunoprecipitation buffer and resuspended in SDS-PAGE sample buffer. Immunoprecipitates were boiled for 5 min and the resulting supernatants were analyzed by immunoblot analysis.

### Immunoblot analysis

Sample protein concentration was determined by BCA protein assay (Thermo Scientific). Equal amounts of protein were resolved by SDS-PAGE and transferred to 0.45 µm nitrocellulose membranes by voltage gradient. The resulting blots were blocked overnight in 5% nonfat dry milk and subsequently probed with primary antibody. The antibodies were used at the indicated dilutions: anti-Ago1 (Cell Signaling. cat #5053) at 1∶1000, anti-Ago2 (Wako, cat# 011-22033) at 1∶1000, anti-HA 6E2 (Cell Signaling, cat # 2367) at 1∶1000, anti-Tubulin (Sigma, cat # T6074) at 1∶1000, anti-Topoisomerase I (Santa Cruz, cat #sc-10783) at 1∶500, anti-RNAP II (Millipore, cat # 05-623) at 1∶5000, anti-Dicer (Santa Cruz, cat #sc-30226) at 1∶1000, anti-Drosha (Cell Signaling, cat #3364) at 1∶1000, and anti-TFIIB (Cell Signaling, cat #4169) at 1∶1000. Immunodetection occurred by incubating blots with appropriate secondary HRP-linked antibodies and utilizing the SuperSignal West Pico Chemiluminescent kit (Thermo Scientific) to visualize antigen-antibody complexes.

### RNAi knockdown

All siRNAs were designed using the BLOCK-iT RNAi Designer Program (Invitrogen). Ago1 knockdown was accomplished by using a pool of 3 siRNAs, while single duplexes were used to knockdown Dicer or Drosha. A pool of 3 non-specific siRNAs served as controls. Transfections were carried out using Lipofectamine RNAiMax (Invitrogen) according to the manufacturer's instructions. All siRNA sequences are listed in Table S16.

### RNA extraction and qRT-PCR analysis

Total RNA was isolated by using the RNeasy Mini Kit (Qiagen). ∼1 µg of total RNA was reverse transcribed into cDNA with MMLV reverse transcriptase (Promega) in conjunction with oligo(dT) primers. The resulting cDNA samples were subjected to real-time PCR analysis using gene-specific primers. All primer sequences are listed in Table S16.

### Chromatin immunoprecipitation

Chromatin immunoprecipitation (ChIP) was performed as previously described with slight modification [11]. Chromatin was prepared from PC-3 cells following crosslinking with formaldehyde. DNA was sheared to an average size of ∼500 bp using the Bioruptor sonicator (Diagenode) set to ‘high’ with 30 sec ON/OFF pulses for 8 min for a total of 8 cycles. Chromatin was immunoprecipitated overnight at 4°C using 5 µg of the following antibodies: anti-Ago1 (Wako, clone 2A7), anti-Ago2 (Wako, clone 2D4), anti-H3K4me3 (Millipore, cat# 07-473), and mouse IgG (Millipore, cat# 12-371). The following day, the samples were incubated with 25 µl Protein G Dynabeads (Invitrogen) for 2 hrs at 4°C. Immunoprecipitates were sequentially washed with low salt, high salt, and TE buffer. Eluates were collected and reverse crosslinked at 65°C overnight. ChIP DNA was treated with Proteinase K, purified with phenol/chloroform, treated with RNase A, and purified using the Qiaquick PCR purification kit (Qiagen). Target amplification and detection was performed by the 7500 Fast Real-Time System (Applied Biosystems). All reactions were prepared in 10 µl volumes containing 2 µl DNA, 2× Fast SYBR Green master mix (Applied Biosystems), and region-specific primer sets (Table S16). Each sample was analyzed in triplicate. Enrichment was determined by using the 2^−ΔCT^ method relative to input DNA or IgG control. Primer specificity was confirmed by evaluating dissociation curves and independently analyzing amplified product on an agarose gel. For Ago ChIP-western analysis, IP was performed essentially the same way as above and the beads were resuspended in 2× SDS sample buffer and boiled for 5 min. Supernatant was collected and analyzed by western blotting analysis.

### Library preparation and ChIP-seq analysis

Each library was prepared by combining the eluates from two ChIP experiments and following the Illumina ChIP-seq library preparation protocol. Briefly, ∼10 ng DNA was end-repaired and subsequently labeled with an additional “A” base on the 3′ ends of the DNA fragments. The resulting DNA samples were ligated to oligonucleotide adaptors and amplified by PCR to construct the individual libraries. Each library was size-selected for DNA fragments ranging between ∼200–300 bp by gel electrophoresis purification. Sample quality was assessed on a Bioanalyzer (Agilent) using the Hypersensitive DNA kit (Agilent) prior to sequencing. Libraries were diluted to 10 nM and sent to the Vincent J. Coates Genomics Sequencing Laboratory at UC Berkeley (http://qb3.berkeley.edu/gsl) for sequencing analysis on a Hiseq2000 Sequencing System (Illumina). Additional detail on ChIP-seq is available in **Text S2**.

### Data access

The ChIP-seq and microarray data from this study have been deposited into the GEO database under the accession numbers GSE40536 and GSE42600.

Other experimental procedures are available in **Text S2**.

## Supporting Information

Figure S1 Cellular distribution of Ago1 and Ago2. (**A**) Distribution of endogenous Ago2 was visualized in PC-3 cells by immunofluorescence using an antibody specific for human Ago2. Distribution of HA-tagged Ago1 (HA-Ago1) or Ago2 (HA-Ago2) was evaluated in stable cell lines using an antibody specific for the HA epitope. Representative immunofluorescent images were taken at 400× magnification. Staining with only secondary antibody control served as a negative (Neg) control. DAPI dye (blue) was used to label nuclei in the merged (Merge) micrographs. (**B**) PC3 cells were transiently transfected with GFP-tagged Ago1 (GFP-Ago1) or Ago2 (GFP-Ago2) overexpression constructs. Immunofluorescent images were taken 3 days following transfection.
(JPG)

Figure S2 Negative controls for Ago1 and Ago2 staining in parental PC-3 cells and PC-3 cells expressing HA-tagged Ago1 or Ago2. Purified nuclei from stable cell lines expressing HA-Ago1 (PC3-HA-Ago1), HA-Ago2 (PC3-HA-Ago2) or parental PC-3 cells were analyzed by IF staining omitting the primary antibody (anti-HA or anti-Ago). To ensure the specificity of the anti-HA antibody, HA staining was performed on nuclei from PC-3 cells without expressing the HA tagged Agos. DAPI (blue) was used to counterstain nuclei. Representative immunofluorescent images were taken at 1000× magnification (scale bar: 10 µm).
(JPG)

Figure S3 Confirmation of RNAP II co-immunoprecipitation. Immunoprecipitation (IP) assays were performed on nuclear extracts from PC-3 cells using an antibody against RNAP II. IgG served as a negative IP control. Immunoprecipitates were analyzed by immunoblotting (IB) with anti-TFIIB, which served as a positive control for RNAP II co-IP as described in Figure 2. % Input represents % nuclear extract used.
(JPG)

Figure S4 Depletion of mature miRNA levels by Dicer or Drosha knockdown. (**A and B**) PC-3 cells were transfected with 50 nM concentrations of siControl, siDicer, or siDrosha for 3 days. Knockdown efficiency of each siRNA was determined by qRT-PCR using primer sets specific for Dicer or Drosha. Expression levels (mean ± SD from 3 independent experiments) were normalized to GAPDH relative to siControl treatments. (B)Knockdown efficiency was further confirmed by western blotting analysis using antibodies specific for Dicer or Drosha (**C**) Perturbation in miRNA biogenesis following Dicer or Drosha knockdown was determined by evaluating expression levels of several highly expressed miRNAs following siRNA treatment. Relative expression of mature miR-744, miR-19a, and miR-19b were determined by miRNA-specific qRT-PCR. Values were normalized to snU6 relative to siControl treatments (n = 2). (**D**) miRNA levels were reduced in HCT116-Dicer Exon5 KO cells compared to the parental cells (WT). Relative expression of mature miR-744, miR-19a, and miR-19b were determined as in (C).
(JPG)

Figure S5 ChIP-seq antibody specificity. (**A**–**C**) Immunoprecipitation (IP) assays were performed on PC-3 cells stably overexpressing HA-tagged eGFP (HA-eGFP), Ago1 (HA-Ago1), or Ago2 (HA-Ago2) using an antibody specific to the HA epitope. The resulting immunoprecipitates were immunoblotted (IB) for HA (**A**), Ago1 (**B**) and Ago2 (**C**) using antibodies that recognize HA, Ago1 and Ago2 respectively. No cross-reactivity was detected by immunoblot (IB) analysis using either the Ago1- or Ago2-specific antibodies. (**D**) Validation of Ago1 antibody specificity in ChIP. ChIP-Western analysis was performed on crosslinked chromatin from PC-3 cells using anti-Ago1 (2A7; Wako) in the IP step followed by IB analysis. IgG was used as a negative control.
(JPG)

Figure S6 ChIP-seq quality control, peak validation and peak size distribution. (**A**) Chromatin was immunoprecipitated with antibodies specific to H3K4me3, Ago1, or Ago2. Each isolated library was size-selected for DNA fragments ranging between ∼200–300 bp. Overall range was confirmed by electrophoresis on a Bioanalyzer prior to DNA sequencing. Input corresponds to the control library generated from total genomic DNA. (**B**) Independent ChIP experiments were performed in PC-3 cells to validate ChIP-seq data on 27 randomly selected regions containing Ago1 peaks. Shown is fold enrichment of Ago1 relative to the IgG control (IP/IgG) for each primer set grouped within different FDR ranges (mean ± SD of two independent experiments). Primers were designed to flank CCAT called peaks. A region devoid of Ago1 binding was amplified as a negative (Neg) control. (**C**) The table summarizes the validation rate for each FDR range evaluated in (B). Highlighted in yellow indicates the FDR range containing the selected FDR cutoff value of 0.054. (**D**) Independent ChIP analyses were performed to validate ChIP-seq data at four genes (RRM1, PIK3CA, CDC6, and PRKCH) with H3K4me3 and Ago1 peaks at their TSSs. % Input was determined by qPCR using primers designed within the ChIP-seq peaks (mean ± SD of two independent experiments). IgG was used as a negative control. (**E** and **F**) Size distribution of Ago1 and H3K4me3 peaks.
(JPG)

Figure S7 Distribution of Ago1 peaks on chromosomes. Shown is Ago1 peak density (peaks/10 kb) on all human chromosomes in comparison to other chromosomal features including gene density (genes/10 kb), percent GC content (GC%), and percent repetitive sequence composition (Repeat%).
(JPG)

Figure S8 Validation of gene expression profiling data. (**A**) PC-3 cells were mock transfected or transfected with siControl or siAgo1 at 10 nM for 48 hrs. Knockdown efficiency was evaluated by qRT-PCR and immunoblot analysis. (**B**) Microarray data following Ago1 knockdown (siAgo1) was plotted in a volcano plot relative to control treatments (siControl+Mock). Red and blue dots denote genes significantly (*P*<0.05) up- or downregulated, respectively. (**C** and **D**) Relative expression of selected down- (C) and upregulated (D) genes was validated by qRT-PCR (mean ± SD from 2 independent experiments). Values were normalized to GAPDH and plotted as log2 fold change to the average of mock and siControl treatments. Fold change values from microarray experiment are also included in the plots for comparison. Pearson correlation coefficient with *P* value between microarray and qPCR values is shown.
(JPG)

Figure S9 Cytoband enrichment of AbG-5 kb genes. Indicated are the top 20 enriched cytobands for AbG-5 kb genes as determined by using the DAVID bioinformatics tool.
(JPG)

Figure S10 Pathway enrichment of Ago1-bound genes. (**A, B**) Indicated are the top 30 enriched pathways (*P*<0.01) for the AbGs-1 kb (A) and AbGs-0.5 kb (B) gene groups as determined by KEGG pathway enrichment analysis. (**C**) Shown is the top 21 enriched non-redundant biological processes (*P*<7.6×10^−7^) in the AbG-5 kb gene set. Gene ontology (GO) enrichment analysis was performed to identify overrepresented biological processes by using the DAVID bioinformatics tool.
(JPG)

Figure S11 Gene category enrichment of up- and downregulated ArGs. (**A, B**) Indicated are the top 20 enriched non-redundant gene ontology (GO) categories in up- (A) and downregulated (B) genes following Ago1 depletion. GO enrichment analysis was performed by using the DAVID bioinformatics tool. Note downregulated ArGs have smaller *p*-values than upregulated ArGs.
(JPG)

Figure S12 Ago1 is overexpressed in cancerous prostate cells relative to non-cancerous prostate cells and its depletion inhibits cell cycle. (**A**) Whole cell lysate (WCL) was isolated from the indicated cell lines and subject to immunoblot analysis using antibodies specific to Ago1 or Tubulin. Detection of Tubulin served as a protein loading control. N.C. Non-malignant cell lines. (**B**) PC-3 cells were transfected with siControl or siAgo1 for 72 days and analyzed by flow cytometry after PI staining to measure DNA content. Shown are examples of resulting FL2A histograms. (**C**) Flow cytometry data was analyzed to determine cell cycle distribution (mean ± SD of two independent experiments). Percentages correspond to the amount of cells present in the treatment populations at the indicated phases of cell cycle (G1, S, or G2/M).
(JPG)

Table S1 Summary of ChIP-seq genome alignment.
(XLSX)

Table S2 Summary of ChIP-seq results.
(XLSX)

Table S3 List of Ago1 peaks (FDR 0.054).
(XLSX)

Table S4 List of H3K4me3 peaks (FDR 0.054).
(XLSX)

Table S5 Ago1 peak distribution on chromosomes.
(XLSX)

Table S6 Ago1 peak distribution on chromosome correlates with gene density.
(XLSX)

Table S7 Genes downregulated by Ago1 knockdown (>1.2 fold, P<0.05).
(XLSX)

Table S8 Genes upregulated by Ago1 knockdown (>1.2 fold, P<0.05).
(XLSX)

Table S9 Enriched miRNAs with predicted targets in Ago1-bound sequences.
(XLSX)

Table S10 Ago1-bound 5 kb genes (AbGs-5 kb).
(XLSX)

Table S11 Ago1-bound 1 kb genes (AbGs-1 kb).
(XLSX)

Table S12 Ago1-bound 0.5 kb genes (AbGs-0.5 kb).
(XLSX)

Table S13 Enrichment cytobands in AbGs-1 kb genes.
(XLSX)

Table S14 Enrichment cytobands in AbGs-0.5 kb genes.
(XLSX)

Table S15 Enrichment of Ago1 bound/regulated genes in Cancer Pathways.
(XLSX)

Table S16 Sequences for siRNAs and oligonucleotide primers.
(XLSX)

Text S1 Overlapping of Ago1 peaks with genome “HOT” regions.
(PDF)

Text S2 Supplemental Methods. Alignment of ChIP-seq reads to the human genome. ChIP-seq peak calling. ChIP-seq peak validation. Additional bioinformatics analyses. cDNA microarray. Microarray data analysis. Pathway/cytoband enrichment analyses. Quantification of miRNA expression. Cell Cycle Analysis. Supplemental References.
(PDF)
