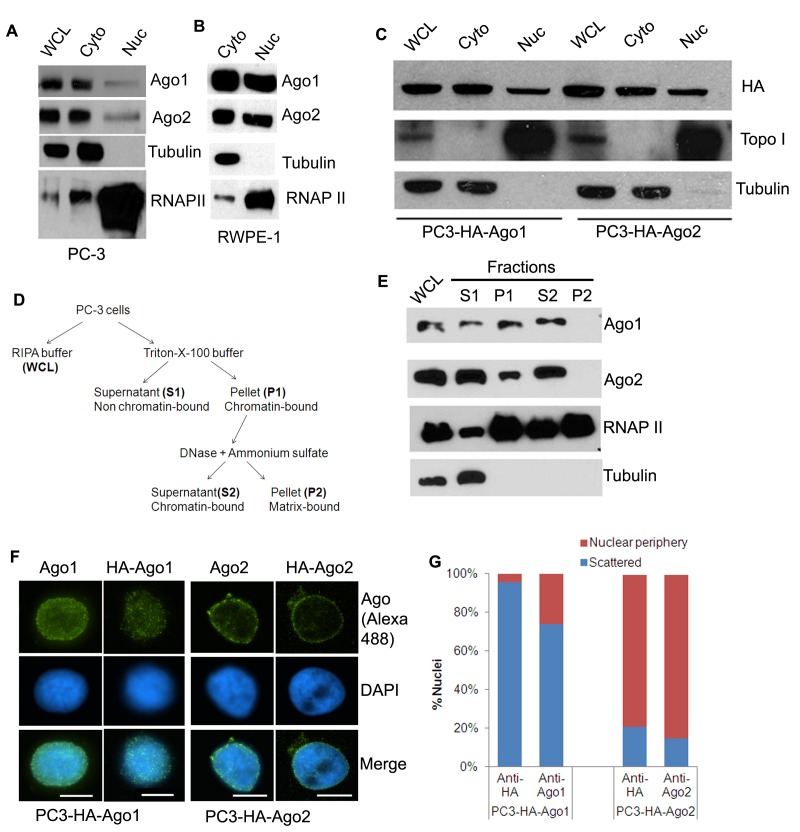# Correction: Ago1 Interacts with RNA Polymerase II and Binds to the Promoters of Actively Transcribed Genes in Human Cancer Cells

**DOI:** 10.1371/annotation/e7c49fcc-0bc2-4aa9-bbcf-f4b386eb2cd0

**Published:** 2014-01-06

**Authors:** Vera Huang, Jiashun Zheng, Zhongxia Qi, Ji Wang, Robert F. Place, Jingwei Yu, Hao Li, Long-Cheng Li

In Figure 1A, the panel showing Ago1 protein levels is incorrectly a duplicate of the panel showing Ago2 protein levels. The authors apologize for this error, which did not exist in an earlier version of the figure and was accidentally introduced during reorganization of the figure when it was prepared for submission to PLOS Genetics. Please see the correct Figure 1 here: 

**Figure:**