# Supplementary material for: Ago1 Interacts with RNA Polymerase II and Binds to the Promoters of Actively Transcribed Genes in Human Cancer Cells
Source: PLoS Genet. 2013 Sep 26;9(9):e1003821. doi: 10.1371/journal.pgen.1003821 (PMC3784563; doi:10.1371/journal.pgen.1003821)
Supplement: Text S1 — Overlapping of Ago1 peaks with genome “HOT” regions. (PDF) [file pgen.1003821.s029.pdf]

Supplemental information

Ago1 interacts with RNA polymerase II and binds to the promoters of actively transcribed genes in human cancer cells

Vera Huang[1*], Jiashun Zheng[2*], Zhongxia Qi[3], Ji Wang[1], Robert F. Place[1], Jingwei Yu[3], Hao Li[2§], and Long-Cheng Li[1§]

[1]Department of Urology and Helen Diller Family Comprehensive Cancer Center, University of California San Francisco, San Francisco, CA 94158, USA

[2]Department of Biochemistry and Biophysics, University of California San Francisco, San Francisco, CA 94158, USA

[3]Department of Laboratory Medicine, University of California San Francisco, San Francisco, CA 94107, USA

*These authors contributed equally to this work

[§] To whom correspondence should be addressed: L.C.L, E-mail: lilc@urology.ucsf.edu or H. L, E-mail: haoli@genome.ucsf.edu

Text S1. Supplemental Text

Minimal overlap between Ago1 peaks and "HOT" regions. To rule out the possibility that Ago1 binding events we observed from ChIP-seq were just a manifestation of so-called "HOT" regions, genomic loci highly co-bound by transcription factors, we compared the Ago1 peaks from our results with the "HOT" regions identified in the paper by Yip et al. [24]. By analyzing the ENCODE data derived from 5 different human cell types for 117 transcription related factors , Yip et al found that "HOT" regions accounted for 0.83% ~ 0.86% of whole genome size in each cell type. We calculated overlaps of Ago1 peaks derived from PC-3 cells with all "HOT" regions consolidated from 5 different cell types. We define an overlap between an Ago1 peak and a HOT region as having at least 50% overlap covered by the smaller region (usually the HOT regions). We found that only 33% of the Ago1 peaks overlapped with the combined "HOT" regions and the majority (67%) of the Ago1 peaks were not localized within these "HOT" regions. Although "HOT" regions are considered cell type specific [24], the use of "HOT" regions pooled from 5 different cell types in our comparison may have exaggerated the degree of overlap. Therefore, our analysis suggests that "HOT" regions may only explain a small fraction of the Ago1 binding while majority of Ago1 binding events do not coincide with "HOT" regions.
