# Supplementary material for: Ago1 Interacts with RNA Polymerase II and Binds to the Promoters of Actively Transcribed Genes in Human Cancer Cells
Source: PLoS Genet. 2013 Sep 26;9(9):e1003821. doi: 10.1371/journal.pgen.1003821 (PMC3784563; doi:10.1371/journal.pgen.1003821)
Supplement: Text S2 — Supplemental Methods. Alignment of ChIP-seq reads to the human genome. ChIP-seq peak calling. ChIP-seq peak validation. Additional bioinformatics analyses. cDNA microarray. Microarray data analysis. Pathway/cytoband enrichment analyses. Quantification of miRNA expression. Cell Cycle Analysis. Supplemental References. (PDF) [file pgen.1003821.s030.pdf]

Supplemental information

Ago1 interacts with RNA polymerase II and binds to the promoters of actively transcribed genes in human cancer cells

Vera Huang[1*], Jiashun Zheng[2*], Zhongxia Qi[3], Ji Wang[1], Robert F. Place[1], Jingwei Yu[3], Hao Li[2§], and Long-Cheng Li[1§]

[1]Department of Urology and Helen Diller Family Comprehensive Cancer Center, University of California San Francisco, San Francisco, CA 94158, USA

[2]Department of Biochemistry and Biophysics, University of California San Francisco, San Francisco, CA 94158, USA

[3]Department of Laboratory Medicine, University of California San Francisco, San Francisco, CA 94107, USA

*These authors contributed equally to this work

[§] To whom correspondence should be addressed: L.C.L, E-mail: lilc@urology.ucsf.edu or H. L, E-mail: haoli@genome.ucsf.edu

Text S2. Supplemental Methods

Alignment of ChIP-seq reads to the human genome. Following base-calling by CASAVA (v1.8.0), we used bowtie [48] to align sequence reads to the human genome (hg19). Default parameters were used when running bowtie, which allow only 2 mismatches in the first 28 seed bases. For reads aligned to multiple positions, only one alignment was reported in the output (bowtie -m 1).

ChIP-seq peak calling. Peaks were identified in ChIP-Seq data by using CCAT (control-based ChIP-Seq analysis tool) [23] on raw reads that uniquely mapped back to the human genome. Reads from input DNA were used as background to control for sequencing bias caused by region-specific effects or copy number variation. Authentic peaks were designated by setting the FDR (false discovery rate) threshold to ≤0.054. Raw ChIP-seq reads and peak calling data have been deposited into GEO under the accession number GSE40536.

ChIP-seq peak validation. 27 randomly selected Ago1 peaks with FDRs ranging from 0.001 to 0.152 were chosen to validate ChIP-seq data and define the FDR cutoff value. Independent ChIP experiments were performed to evaluate Ago1 occupancy at the 27 selected sites using region-specific primer sets in conjunction with real-time PCR. Refer to the ‘Chromatin Immunoprecipitation’ protocol in the Materials and Methods section

for further detail on enrichment calculations. All ChIP-seq validation primers are listed in Table S16.

Additional bioinformatics analyses.

General statistics of Ago1 peaks: For each chromosome, the number of Ago1 peaks with FDR ≤0.054 and the number of genes (unique Ensemble GeneIDs) were counted. This information along with % GC content (GC%) and % repetitive sequences (Repeat%) are listed for each chromosome in Table S5.

Peak distribution with regards to genic features: We used the Cis-regulatory Element Annotation System (CEAS) tool [49] on Ago1 peaks to determine enrichment of any genic features (i.e. promoters, gene bodies, and 3' flanking regions) with respect to the genome background.

Repetitive element analysis of Ago1 peaks: The Pre-Masked Human Genome (HG19 with Repeat library 20120124) was downloaded from the RepeatMasker website (http://www.repeatmasker.org). We searched the pre-masked genome to identify all repetitive regions overlapped with the Ago1 peaks. The total number of base pairs for each repeat family were calculated and compared to whole genome background.

microRNA target prediction: Miranda [50] was used to predict putative miRNA target sites in the Ago1-bound peak sequences for all human miRNA listed in the miRbase database [51]. For each Ago1 peak analyzed, we also randomly sampled 5 genomic sequences of the same length and GC content from the same chromosome. These fragments were combined into a single sequence and scanned for putative miRNA target sites to serve as a comparative control. For a given miRNA (*i*), the expected number of target sites (N_i) were calculated by dividing the number of predicted targets for *i* in the control sequence (C_i) by 5: $N_i = C_i/5$. The fold enrichment of predicted target sites for a given miRNA (*i*) in Ago1 peaks were calculated with the equation $M_i = n_i/N_i$ in which n_i is the number of target sites for the miRNA(*i*) in the Ago1 peak. Distribution of target site enrichment for all miRNAs is shown in Figure 6A. The long tails correspond to miRNAs with putative target sites enriched ($M_i>1$ or $\log_2 (M_i)>0$) or depleted ($M_i<1$ or $\log_2 (M_i)<0$) in Ago1 peaks. Based on distribution, we chose $M_i = \pm1.5$ as a cutoff to define miRNAs with enriched ($M_i>1.5$) or depleted ($M_i<-1.5$) target prediction in Ago1 peaks. *P*-values were calculated with Poisson cumulative distribution in which N_i served as the expected value of target sites. The sequences of enriched miRNAs were analyzed to identify any 6- or 7-mer sequences overrepresented as compared to non-enriched miRNAs. The number of the occurrences for all 6- and 7-mers identified in the enriched miRNAs was compared to the corresponding number found in the non-enriched miRNAs. Those 6- and 7-mers that were significantly overrepresented in the enriched miRNAs were compared to *de novo* motif analysis by MEME Suite [52] in the enriched miRNA sequences.

Distance of Ago1 peaks to nearest TSS: For each Ago1 peak, we identified the nearest transcription start site (TSS) on both sides of the peak. The distance between the peak summit and TSS was calculated with a minus or plus sign corresponding to whether the

Ago1 peak was located upstream or downstream of the nearest TSS, respectively. For all peaks, distances to the nearest TSS were compiled into a single distribution histogram (Figure 4D).

Enrichment of ArGs in AbG groups following siAgo1 treatment: We used several TSS distance thresholds (i.e. 0.5 kb, 1.0 kb, and 5.0 kb) to generate AbGs (Ago1-bound gene) sets based on distance between Ago1 peaks and TSSs. We computed the frequencies of up- and downregulated ArGs in genes measured by microarray: $p_{up} = {N_{up}}/{N_{microarray}}$ and $p_{down} = {N_{down}}/{N_{microarray}}$, where N_{up} and N_{down} are number of up- and down regulated genes identified by microarray respectively; $N_{microarray}$ is the number of all genes covered in the microarray. For each AbG set, we counted the number of overlapping up- or downregulated ArGs (Ago1-responsive genes) as determined by microarray analysis following Ago1 knockdown ($O_{up,s}$ and $O_{down,s}$ for the observed numbers in the AbGs set *S*). p_{up} and p_{down} were used to calculate the expected number of genes to be deregulated in the AbGs fraction represented within the array: $E_{up,s} = N_s p_{up}$ and $E_{down,s} = N_s p_{down}$, where N_s is the number of genes covered by microarray in each AbGs set *S*. *P*-values for enrichment of ArGs in each set of AbGs were calculated as the probability of getting $O_{up,s}$ (or $O_{down,s}$) or more changed genes in a total N_s genes with binomial distribution using p_{up} and p_{down} as background frequency: $P_{up,s} = 1 - \sum_{i=0}^{O_{up}-1} \binom{N_s}{i} p_{up}^i (1-p_{up})^{N_s - i}$ and $P_{down,s} = 1 - \sum_{i=0}^{O_{down}-1} \binom{N_s}{i} p_{down}^i (1-p_{down})^{N_s - i}$ (Figure 5B).

Dependency of gene expression changes on the distance of Ago1 peaks from TSS: Enrichment of ArGs within the AbG set were evaluated using a slideing window to determine the statistical dependency of changes in gene expression on the distance between their Ago1 peaks and TSSs. Genes (*g*) were first sorted by the distance between their TSS and nearby Ago1 peaks: $\{g_1, g_2, ... g_n\}$ in which $TSS_i \le TSS_{i+1}$ (TSS_i is the distance of gene *i* to its nearby Ago1 peak, *n* is the total number of AbGs within 5kb of the Ago1 peaks). We defined *n-1000+1* set of AbGs: $S_i = \{g_{i,} g_{i+1}, ... g_{i+999}\}$. For each set of genes, the median value (M_i) of the $\{TSS_{i,} TSS_{i+1}, ... TSS_{i+999}\}$ was calculated (by the definition of the gene set, we always have $M_i \le M_{i+1}$). The enrichment p-values for the up-regulated and down-regulated ArGs within each AbGs set (S_i) were then calculated using binomial distribution with similar formula defined above. For visualization, the *P* values of the enrichments were plotted against M_i (Figure 5C).

cDNA microarray. PC-3 cells were transfected in duplicate with a pool of 3 siRNAs targeting Ago1 (siAgo1) or a pool of 3 non-specific siRNAs (siControl) at 10 nM concentrations for 48 hrs. Mock samples were transfected in absence of siRNA and

served as additional controls. The siAgo1 pool achieved ≥80% knockdown of Ago1 by 48 hrs (Figure S5A). Total RNA was extracted from cells using the RNeasy kit (Qiagen) according to the manufacturer's instructions. Reverse transcription reactions were performed using the Ovation® Pico WTA System V2 (NuGen) to generate cDNA. Fragmentation and labeling was carried out by using the Encore^{TM} Biotin Module (NuGen) to generate biotinylated probes for hybridization to GeneChip® Human Gene 1.0 ST Arrays (Affymetrix). Hybridization, washing, and scanning were performed according to the GeneChip® Expression Analysis Technical Manual (rev. 3). Microarray data has been deposited into GEO under the accession number GSE42600.

Microarray data analysis. For pairwise analysis, mock and siControl data were combined and compared to siAgo1 treatment results to identify differentially expressed genes. Unpaired *t*-test followed by Benjamini and Hochberg multiple testing correction was applied to determine significance for changes in gene expression. Changes >1.2-fold with *p*-values <0.05 were considered statistically significant. To assess whether a particular pathway may be deregulated following Ago1 depletion, gene ontology (GO) analysis was applied to genes significantly up- and downregulated by siAgo1 treatment.

Pathway/cytoband enrichment analyses. All pathway/cytoband analyses were performed using the DAVID bioinformatics tool (http://david.abcc.ncifcrf.gov). The default DAVID human genome genes were used as the background gene population for ChIP-seq data, whereas the Uniset Human 20K genes were used as background for microarray analysis. *P* values presented are EASE score which are modified Fisher's Exact P values [53] unless otherwise indicated.

Quantification of miRNA expression. Total RNA was extracted using the miRNeasy Mini Kit (Qiagen) according to the manufacturer's protocol. Reverse transcription reactions containing 200 ng of total RNA were performed using the TaqMan® MicroRNA Reverse Transcription Kit (Applied Biosystems, Foster City, CA) in conjunction with miRNA-specific primers. Real-time PCR was carried out using Taqman® MicroRNA Assay kits specific to human miR-744, miR-19a, or miR-19b in order to quantify miRNA expression levels. Amplification of snU6 served as an endogenous control to normalize data. Each sample was analyzed in triplicate.

Cell Cycle Analysis. Transfected cells were trypsinized, washed with PBS, and counted by trypan blue staining. Approximately 1.0×10^6 cells were resuspended in 70% ethanol and fixed at 4°C for 1 hr. Cells were pelleted and stained in 1 ml of Krishna's buffer (0.1% sodium citrate, 0.02 mg/ml RNase A, 0.3% Triton X-100, and 0.05 mg/ml PI, pH 7.4) for 1 hr at 4°C. Samples were centrifuged, washed with PBS buffer, and analyzed by flow cytometry on a FACSCalibur flow cytometer (BD Bioscience). A total of 10,000 events were collected and staining intensity was analyzed using the FL2 channel for relative DNA content. Forward and side scatter gates and a doublet discrimination plot was set to include whole and individual cell populations, respectively. The resulting data was analyzed by using the Modfit LT program (Verity Software House) to determine cell cycle distribution.

Supplemental References

48. Langmead B, Trapnell C, Pop M, Salzberg SL (2009) Ultrafast and memory-efficient alignment of short DNA sequences to the human genome. Genome Biol 10: R25.
49. Shin H, Liu T, Manrai AK, Liu XS (2009) CEAS: cis-regulatory element annotation system. Bioinformatics 25: 2605-2606.
50. Enright AJ, John B, Gaul U, Tuschl T, Sander C, et al. (2003) MicroRNA targets in Drosophila. Genome Biol 5: R1.
51. Kozomara A, Griffiths-Jones S (2011) miRBase: integrating microRNA annotation and deep-sequencing data. Nucleic Acids Res 39: D152-157.
52. Bailey TL, Boden M, Buske FA, Frith M, Grant CE, et al. (2009) MEME SUITE: tools for motif discovery and searching. Nucleic Acids Res 37: W202-208.
53. Hosack DA, Dennis G, Jr., Sherman BT, Lane HC, Lempicki RA (2003) Identifying biological themes within lists of genes with EASE. Genome Biol 4: R70.
